# Optimal Parameter Design of Coarse Alignment for Fiber Optic Gyro Inertial Navigation System

**DOI:** 10.3390/s150715006

**Published:** 2015-06-25

**Authors:** Baofeng Lu, Qiuying Wang, Chunmei Yu, Wei Gao

**Affiliations:** 1 College of Automation, Harbin Engineering University, Harbin 150001, China; E-Mail: gaow@hrbeu.edu.cn; 2 College of Information and Communication Engineering, Harbin Engineering University, Harbin 150001, China; E-Mail: wqy869087@163.com; 3 Aerospace and Materials Engineering, National University of Defense Technology, Changsha 410000, China; E-Mail: chunmeiyu2014@163.com; 4 National Aerospace Intelligence Control Technology Laboratory, Beijing Aerospace Automatic Control Institute, Beijing 100854, China

**Keywords:** fiber optic gyro (FOG), inertial navigation system (INS), coarse alignment algorithm, optimal parameter design

## Abstract

Two different coarse alignment algorithms for Fiber Optic Gyro (FOG) Inertial Navigation System (INS) based on inertial reference frame are discussed in this paper. Both of them are based on gravity vector integration, therefore, the performance of these algorithms is determined by integration time. In previous works, integration time is selected by experience. In order to give a criterion for the selection process, and make the selection of the integration time more accurate, optimal parameter design of these algorithms for FOG INS is performed in this paper. The design process is accomplished based on the analysis of the error characteristics of these two coarse alignment algorithms. Moreover, this analysis and optimal parameter design allow us to make an adequate selection of the most accurate algorithm for FOG INS according to the actual operational conditions. The analysis and simulation results show that the parameter provided by this work is the optimal value, and indicate that in different operational conditions, the coarse alignment algorithms adopted for FOG INS are different in order to achieve better performance. Lastly, the experiment results validate the effectiveness of the proposed algorithm.

## 1. Introduction

The Fiber Optic Gyro (FOG) Inertial Navigation System (INS) is a modern system that has removed most of the mechanical complexity of platform systems by having the sensors attached rigidly or “strapped down” to the body of the host vehicle [1]. It belongs to Strapdown Inertial Navigation System (SINS) and has been substituted for platform INS [1,2]. In the normal course of FOG INS operation, the system’s initial attitude needs to be determined [3,4], and the attitude determination is achieved by the alignment process. Normally, the alignment process is divided into two steps, *i.e.*, coarse and fine alignment, with coarse alignment followed by fine alignment [5]. The purpose of coarse alignment is to provide a good initial attitude for the fine alignment process. In this way, the duration of the total alignment process can be made shorter [6].

For the coarse alignment there is no *a priori* knowledge of initial conditions [7]. Only the measurement information from accelerometers and FOG outputs can be used. This fact forces the development of a non-linear alignment algorithm, and analytic methods are generally used for coarse alignment. In the ground base, the attitude can be determined directly by the analytic coarse alignment method using the gravity and earth rotation vectors [8]. Normally, the accuracy of this method can meet the requirement of fine alignment under the disturbance of limited vibration. However, FOG INS is usually applied in complex and volatile environments, and then the system has to withstand random movements which may be violent, such as a ship’s pitch and roll [9]. The ground coarse alignment techniques, henceforth, can not be used, since the measurement of the earth rotation rate provided by FOG is disturbed by high rotational values (several orders of magnitude greater than the earth rotation rate) [10].

In order to resolve this problem, a new analytic coarse alignment method based on the inertial reference frame for FOG INS has been provided [11]. This method is developed based on the fact that the gravity expressed in inertial space defines a cone whose main axis is the rotational axis of the Earth. Many researchers have investigated this topic, mainly based on the structure of noncollinear vectors [9,12,13]. In Reference 9, the noncollinear vectors are constructed by a velocity vector that is determined by gravity vector integration. In Reference 12, the authors provided a construction method by which the noncollinear vectors are acquired by a position vector that is produced by velocity vector integration (obtained by gravity integration). All of them have a lack of rigorous selection of integration time, so the performance of the coarse alignment algorithms may not be optimal.

In order to give a criterion for selecting the integration time, and make the selection more accurate, the optimal parameter design of coarse alignment algorithms for FOG INS is done in this paper. First, the analysis of these two algorithms is made, and it is focused on the quasi-stationary conditions. Then with the analysis of the error characteristics, the optimal parameter design of these two algorithms is derived. Finally, based on the analysis and optimal parameter design, the adequate selection of the most accurate algorithm for FOG INS according to the actual operational conditions is provided. The remainder of this paper is organized as follows: the coordinate frames used in this paper are addressed in Section 2. In Section 3, the principle of the new analytic coarse alignment method for FOG INS is introduced. Then the algorithms produced by the two different constructions are presented in Section 4. In Section 5, the processes of the error analysis and optimal parameter design are performed. Finally, in Section 6 and Section 7, simulation and experiment results verify the analysis made in Section 5, and Section 8 concludes this paper.

## 2. Coordinate Frame Definitions

The coordinate frames used in this paper are defined as follows:

(1) The b frame is the body coordinate frame. The $x_{b}$ axis is parallel to the vehicle's lateral axis and points to the right. The $y_{b}$ axis is parallel to the vehicle's longitudinal axis and points to forward. The $z_{b}$ axis is parallel to the vehicle's vertical axis and points upward.
(2) The i frame is the non-rotating inertial coordinate frame. It is formed by fixing the earth-fixed coordinate frame at the beginning of the coarse alignment.
(3) The $i_{b}$ frame is the body inertial coordinate frame. It is formed by fixing the b frame at the beginning of the coarse alignment.
(4) The n frame is the navigation frame, used for navigation and attitude representation. In this work, we choose the local level geographic coordinate frame as the n frame.

## 3. Description of General Requirements

The purpose of initial alignment for FOG INS is to determine the direction cosine matrix (DCM), which relates vectors in the body coordinate frame b to the same vectors expressed in the navigation coordinate frame n. Analytically, the DCM could be described as follows using the product chain rule:

(1) $$C_{b}^{n}(t)=C_{i}^{n}(t)C_{i_{b}}^{i}C_{b}^{i_{b}}(t)$$

where $C_{b}^{i_{b}}(t)$ is the transformation matrix that transforms vectors from frame b to frame $i_{b}$ and can be calculated using the FOG output as described in the next section, $C_{i}^{n}(t)$ is given as follows:

(2) $$C_{i}^{n}(t)=\left[\begin{array}{ccc} -\sin\omega_{ie}(t-t_{0}) & \cos\omega_{ie}(t-t_{0}) & 0\\ -\sin L\cos\omega_{ie}(t-t_{0}) & -\sin L\sin\omega_{ie}(t-t_{0}) & \cos L\\ \cos L\cos\omega_{ie}(t-t_{0}) & \cos L\sin\omega_{ie}(t-t_{0}) & \sin L \end{array}\right]$$

where $\omega_{ie}$ is the earth rate, L represents the local latitude, and $t_{0}$ is the start time of the coarse alignment.

Then the task of DCM determination is transformed into the determination of remainder matrix $C_{i_{b}}^{i}$. This matrix can be directly computed using the knowledge of two noncollinear vectors in the two frames (inertial frame i and body inertial frame $i_{b}$). Let a and b represent these two noncollinear vectors, and define their components in the inertial frame as:

(3) $$a^{i}={\left[\begin{array}{ccc} a_{x} & a_{y} & a_{z} \end{array}\right]}^{T}$$

(4) $$b^{i}={\left[\begin{array}{ccc} b_{x} & b_{y} & b_{z} \end{array}\right]}^{T}$$

The vectors a and b transform according to the following expressions:

(5) $$a^{i_{b}}=C_{i}^{i_{b}}a^{i}$$

(6) $$b^{i_{b}}=C_{i}^{i_{b}}b^{i}$$

If c is defined as

(7) c=a×b

We also have:

(8) $$c^{i_{b}}=C_{i}^{i_{b}}c^{i}$$

From Equations (5), (6), and (8), $C_{i_{b}}^{i}$ could be calculated by the following equation:

(9) $$C_{i_{b}}^{i}={\left[\begin{array}{c} {\left(a^{i}\right)}^{T}\\ {\left(b^{i}\right)}^{T}\\ {\left(c^{i}\right)}^{T} \end{array}\right]}^{-1}\cdot \left[\begin{array}{c} {\left(a^{i_{b}}\right)}^{T}\\ {\left(b^{i_{b}}\right)}^{T}\\ {\left(c^{i_{b}}\right)}^{T} \end{array}\right]$$

Thus, by substituting the remainder matrix calculated by Equation (9) in Equation (1), the DCM can be uniquely determined. At this time, the problem of the alignment of FOG INS is basically that of determining the two noncollinear vectors a and b.

**Figure 1:**
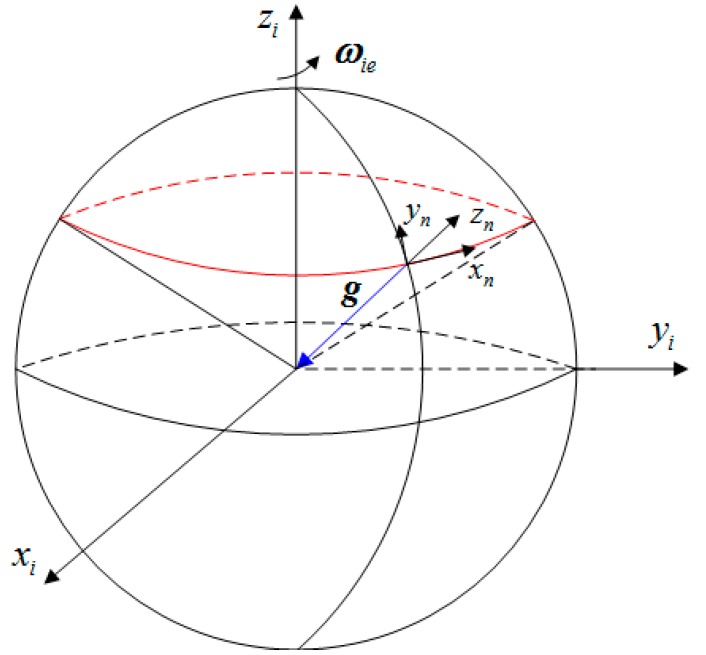
The moving trajectory of gravity in the inertial space.

From Figure 1, it is easy to see that the gravity expressed in inertial space defines a cone whose main axis is the rotational axis of the Earth. So, the projections of gravity onto the frames i and $i_{b}$ at different times are noncollinear. Then the two noncollinear vectors used for $C_{i_{b}}^{i}$ computation can be generated by the gravity vector.

## 4. Two Alignment Algorithms

Two alignment algorithms are provided in this section, and the difference between them is the construction of vectors a and b. Let us begin by providing the updated algorithm for $C_{b}^{i_{b}}(t)$, it can be derived by:

(10) $${\dot{C}}_{b}^{i_{b}}(t)=C_{b}^{i_{b}}(t)[{\text{ω}}_{ib}^{b}\times ]$$

where $C_{b}^{i_{b}}(t_{0})=I$, $\left[{\text{ω}}_{ib}^{b}\times \right]$ denotes that a skew-symmetric matrix function of ${\text{ω}}_{ib}^{b}$, ${\text{ω}}_{ib}^{b}$ is the angular rate of the b frame with respect to the i frame, which can be measured by FOG.

The noncollinear vectors of the two alignment algorithms in frame i are defined in Table 1.

**Table 1:** Noncollinear vectors in inertial frame i.

Alignment Algorithms	Noncollinear Vectors	
	$a^{i}$	$b^{i}$
Algorithm 1	$V^{i}(t_{k1})=-\int_{t_{0}}^{t_{k1}}g^{i}(t)dt$	$V^{i}(t_{k2})=-\int_{t_{0}}^{t_{k2}}g^{i}(t)dt$
Algorithm 2	$S^{i}(t_{k1})=\int_{t_{0}}^{t_{k1}}V^{i}(t)dt$	$S^{i}(t_{k2})=\int_{t_{0}}^{t_{k2}}V^{i}(t)dt$

where $t_{k2}$ is the end-time of coarse alignment, and $t_{0}<t_{k1}<t_{k2}$, $g^{i}(t)$ corresponds to

(11) $$g^{i}(t)={\left[C_{i}^{n}(t)\right]}^{T}g^{n}$$

where $g^{n}={\left[\begin{array}{ccc} 0 & 0 & -g \end{array}\right]}^{T}$ is the gravity vector expressed in the navigation frame.

Since these vectors are constructed by $g^{i}(t)$, $a^{i}$ and $b^{i}$ are precisely known. On the other hand, $a^{i_{b}}$ and $b^{i_{b}}$ are generated by the measurements of accelerometers and FOG. Without linear motion, the actual specific force $f^{b}$ can be expressed as:

(12) $$f^{b}=-g^{b}(t)=-C_{i}^{b}(t)g^{i}(t)$$

Therefore,

(13) $$C_{b}^{i_{b}}(t)f^{b}=-C_{i}^{i_{b}}g^{i}(t)$$

Alternatively, considering Equations (5) and (6) and Table 1, $a^{i_{b}}$ and $b^{i_{b}}$ are denoted as (Table 2).

**Table 2:** Noncollinear vectors in body inertial frame $i_{b}$.

Alignment Algorithms	Noncollinear Vectors	
	$a^{i_{b}}$	$b^{i_{b}}$
Algorithm 1	$V^{i_{b}}(t_{k1})=\int_{t_{0}}^{t_{k1}}C_{b}^{i_{b}}(t)f^{b}dt$	$V^{i_{b}}(t_{k2})=\int_{t_{0}}^{t_{k2}}C_{b}^{i_{b}}(t)f^{b}dt$
Algorithm 2	$S^{i_{b}}(t_{k1})=\int_{t_{0}}^{t_{k1}}V^{i_{b}}(t)dt$	$S^{i_{b}}(t_{k2})=\int_{t_{0}}^{t_{k2}}V^{i_{b}}(t)dt$

Theoretically, the transformation matrices $C_{i_{b}}^{i}$ obtained by these two algorithms are identical in the ideal situation. However, their error characteristics are not completely identical.

## 5. Error Analysis and Optimal Parameter Design

In the preceding section, no error sources are taken into consideration, but as mentioned earlier, the body inertial frame components of a and b are derived by the measurements of accelerometers and FOG. Therefore, the effect of Inertial Measurement Unit (IMU) sensor errors and base motion should be taken into account. The analysis of error characteristics driven by IMU sensor errors and base motion is done in the following section. On the other hand, since the coarse alignment time $t_{k2}$ is generally fixed, and $t_{k1}$ is adjustable, the optimal ratio between median integration time $\Delta t_{k1}=t_{k1}-t_{0}$ and total integration time $\Delta t_{k2}=t_{k2}-t_{0}$ exists and is derived with the help of error analysis. Also, based on the previous analysis, the adequate selection of the most accurate algorithm for FOG INS according to the actual operational conditions is provided.

In practical implementation, the equation for $C_{i_{b}}^{i}$ should be rewritten in the form:

(14) $${\hat{C}}_{i_{b}}^{i}=G^{-1}\hat{F}$$

where

$$G=\left[\begin{array}{c} {\left(a^{i}\right)}^{T}\\ {\left(b^{i}\right)}^{T}\\ {\left(c^{i}\right)}^{T} \end{array}\right]\text{ and }\hat{F}=\left[\begin{array}{c} {\left({\hat{a}}^{i_{b}}\right)}^{T}\\ {\left({\hat{b}}^{i_{b}}\right)}^{T}\\ {\left({\hat{c}}^{i_{b}}\right)}^{T} \end{array}\right]$$

${\hat{a}}^{i_{b}}$ and ${\hat{b}}^{i_{b}}$ represent computed noncollinear vectors, and are shown in Table 3.

**Table 3:** Computed noncollinear vectors in body inertial frame $i_{b}$.

Alignment Algorithms	Noncollinear Vectors	
	${\hat{a}}^{i_{b}}$	${\hat{b}}^{i_{b}}$
Algorithm 1	${\hat{V}}^{i_{b}}(t_{k1})=\int_{t_{0}}^{t_{k1}}{\hat{C}}_{b}^{i_{b}}(t){\hat{f}}^{b}dt$	${\hat{V}}^{i_{b}}(t_{k2})=\int_{t_{0}}^{t_{k2}}{\hat{C}}_{b}^{i_{b}}(t){\hat{f}}^{b}dt$
Algorithm 2	${\hat{S}}^{i_{b}}(t_{k1})=\int_{t_{0}}^{t_{k1}}{\hat{V}}^{i_{b}}(t)dt$	${\hat{S}}^{i_{b}}(t_{k2})=\int_{t_{0}}^{t_{k2}}{\hat{V}}^{i_{b}}(t)dt$

where ${\hat{C}}_{b}^{i_{b}}(t)$ is calculated by:

(15) $${\dot{\hat{C}}}_{b}^{i_{b}}(t)={\hat{C}}_{b}^{i_{b}}(t)[{\hat{\omega }}_{ib}^{b}\times ]$$

${\hat{\omega }}_{ib}^{b}$ denotes the measurement quantity measured by FOG, and can be expressed as:

(16) $${\hat{\omega }}_{ib}^{b}={\text{ω}}_{ib}^{b}+{\text{ε}}^{b}$$

where ${\text{ε}}^{b}$ is the FOG error. The other measurement quantity ${\hat{f}}^{b}$ can be represented as [14,15]:

(17) $${\hat{f}}^{b}=C_{i_{b}}^{b}(t)[-C_{i}^{i_{b}}g^{i}(t)+{\dot{v}}^{i_{b}}(t)+{\text{ω}}_{ie}^{i_{b}}(t)\times v^{i_{b}}(t)]+\nabla^{b}$$

Neglecting the second order small term, it can be simplified into:

(18) $${\hat{f}}^{b}=C_{i_{b}}^{b}(t)[-C_{i}^{i_{b}}g^{i}(t)+{\dot{v}}^{i_{b}}(t)]+\nabla^{b}$$

where ${\dot{v}}^{i_{b}}(t)$ is the external acceleration caused by linear motion, $\nabla^{b}$ represents the accelerometer error.

### 5.1. Error Sources Analysis

It is quite obvious from Table 1 and Table 3 that the elements of G are precisely known, but $\hat{F}$ contains sensor errors and base motion which are uncertain. The error analysis in this section utilizes perturbation methods, then ${\hat{C}}_{i_{b}}^{i}$ and $\hat{F}$ can be expanded in series and arranged in the following forms:

(19) $${\hat{C}}_{i_{b}}^{i}=C_{i_{b}}^{i}+\delta C_{i_{b}}^{i}$$

(20) $$\hat{F}=F+\delta F$$

where $\delta C_{i_{b}}^{i}$ is the error matrix between ${\hat{C}}_{i_{b}}^{i}$ and $C_{i_{b}}^{i}$ and the matrix δF represents the departure of $\hat{F}$ from F.

Consequently, we have:

(21) $$\delta C_{i_{b}}^{i}=G^{-1}\delta F$$

Equation (21) shows that the error of $C_{i_{b}}^{i}$ is caused by the departure of $\hat{F}$ from F. By comparing Table 2 and Table 3, we find that the matrix δF results from the departure of ${\hat{x}}^{i_{b}}$ from $x^{i_{b}}$ (x=a, b). This means that we can study the error sources by analyzing the departure of ${\hat{x}}^{i_{b}}$ from $x^{i_{b}}$. Consider Equation (18) and the following equation:

(22) $${\hat{C}}_{b}^{i_{b}}(t)=C_{b}^{i_{b}}(t)+\delta C_{b}^{i_{b}}(t)$$

where $\delta C_{b}^{i_{b}}(t)$ is the error matrix caused by the FOG error. ${\hat{C}}_{b}^{i_{b}}(t){\hat{f}}^{b}$ can be expressed as:

(23) $${\hat{C}}_{b}^{i_{b}}(t){\hat{f}}^{b}=-C_{i}^{i_{b}}g^{i}(t)+[{\dot{v}}^{i_{b}}(t)+C_{b}^{i_{b}}(t)\nabla^{b}-\delta C_{b}^{i_{b}}(t)C_{i_{b}}^{b}(t)C_{i}^{i_{b}}g^{i}(t)]$$

where products of error quantities have been neglected. Then the departure of ${\hat{x}}^{i_{b}}$ from $x^{i_{b}}$ (x=a, b) can be obtained and described as follows (for the two alignment algorithms):

(24) $${\hat{V}}^{i_{b}}(t_{x})-V^{i_{b}}(t_{x})=\int_{t_{0}}^{t_{x}}\left[{\dot{v}}^{i_{b}}(t\right)+C_{b}^{i_{b}}(t)\nabla^{b}-\delta C_{b}^{i_{b}}(t)C_{i}^{b}(t)g^{i}(t)]dt$$

(25) $${\hat{S}}^{i_{b}}(t_{x})-S^{i_{b}}(t_{x})=\int_{t_{0}}^{t_{x}}\int_{t_{0}}^{t}\left[{\dot{v}}^{i_{b}}(\tau \right)+C_{b}^{i_{b}}(\tau )\nabla^{b}-\delta C_{b}^{i_{b}}(\tau )C_{i}^{b}(\tau )g^{i}(\tau )]d\tau dt$$

where $t_{x}=t_{k1}\;or\;t_{k2}$. Obviously, this departure is mainly caused by $\nabla^{b}$, $\delta C_{b}^{i_{b}}$, and ${\dot{v}}^{i_{b}}$. It means that sensor errors $\nabla^{b}$, ${\text{ε}}^{b}$, and base motion ${\dot{v}}^{i_{b}}$ are the major error sources of the error matrix $\delta C_{i_{b}}^{i}$.

### 5.2. The Effect of Sensor Errors

First, the effect of sensor errors is analyzed. In our analysis, we assume that the accelerometer errors are basically bias errors and the FOG errors are basically constant drifts. It is well known that the steady-state alignment errors are affected by the sensor errors. In general, the relationships between alignment errors and sensor errors are often expressed in the navigation frame because only the analysis of alignment errors in the navigation frame is meaningful, since the final results of alignment need to be expressed in the navigation frame. For many alignment approaches (for instance, gyrocompass alignment and optimum alignment), the relationships can be written as [16,17,18,19]:

(26) $$\phi_{E}=-\nabla_{N}/g$$

(27) $$\phi_{N}=\nabla_{E}/g$$

(28) $$\phi_{U}=-\varepsilon_{E}/\Omega \cos L+\nabla_{E}\tan L/g$$

where $\phi_{E}$ is the east alignment error, $\phi_{N}$ is the north alignment error, $\phi_{U}$ is the up alignment error, $\nabla_{E}$ represents the east accelerometer error, $\nabla_{N}$ represents the north accelerometer error, and $\varepsilon_{E}$ represents the east FOG error. By utilizing the perturbation method, the correlation between $\delta C_{b}^{n}(t)$ and $\delta C_{i_{b}}^{i}$ in this paper can be described as:

(29) $$\delta C_{b}^{n}(t)=\delta C_{i}^{n}(t)C_{i_{b}}^{i}C_{b}^{i_{b}}(t)+C_{i}^{n}(t)\delta C_{i_{b}}^{i}C_{b}^{i_{b}}(t)+C_{i}^{n}(t)C_{i_{b}}^{i}\delta C_{b}^{i_{b}}(t)$$

where $\delta C_{b}^{n}(t)$ is the error matrix related to $\phi_{E}$, $\phi_{N}$, and $\phi_{U}$, and $\delta C_{i}^{n}(t)$ denotes the error matrix of $C_{i}^{n}(t)$. Since the local latitude L and time t are known, $\delta C_{i}^{n}(t)$ is equal to zero. On the other hand, the error source of Equation (15) is only the FOG error, and the total alignment time is short, so the error matrix $\delta C_{b}^{i_{b}}(t)$ in Equation (29) is small and can be ignored. It should be noted that the error matrix $\delta C_{b}^{i_{b}}(t)$ in Equations (24) and (25) could not be neglected because the magnitude of the product between matrix $\delta C_{b}^{i_{b}}(t)$ and gravity vector $C_{i}^{b}(t)g^{i}(t)$ is considerable.

Considering the previously analysis, Equation (29) is simplified into:

(30) $$\delta C_{b}^{n}(t)=C_{i}^{n}(t)\delta C_{i_{b}}^{i}C_{b}^{i_{b}}(t)$$

Evidently, $\delta C_{b}^{n}(t)$ and $\delta C_{i_{b}}^{i}$ are equivalent and transformational, and then the effect of sensor errors on matrix $\delta C_{i_{b}}^{i}$ can be transformed into the influence of sensor errors on matrix $\delta C_{b}^{n}(t)$. In this paper, the relationships between alignment errors and sensor errors in the navigation frame for the two alignment algorithms are provided and can be expressed as Equations (26) and (27), and then the effect of sensor errors is uncorrelated with the integration time. This conclusion can be drawn from Simulation A, since the effect of sensor errors is deterministic. Both of these two alignment algorithms have the same accuracy under the condition of existing internal sensor errors, and it is verified in Simulation A.

### 5.3. Base Motion Effect and Optimal Parameter Design

Secondly, the effect of base motion is analyzed, and the optimal ratio between $\Delta t_{k1}$ and $\Delta t_{k2}$ is provided. In general, base motion typically falls into two categories. One is the angular motion, and the other is the linear motion. Fortunately, these two alignment algorithms are not influenced by angular motion; this conclusion can be drawn from Equations (24) and (25), and is verified in simulation B. Then the remaining problem is the analysis of the effect of linear motion on matrix $\delta C_{i_{b}}^{i}$, and this problem is resolved by utilizing the property of matrix norm.

Under the disturbance of linear motion, the departure of ${\hat{x}}^{i_{b}}$ from $x^{i_{b}}$ (x=a, b) can be simplified into (for the two alignment algorithms):

(31) $${\hat{V}}^{i_{b}}(t_{x})-V^{i_{b}}(t_{x})=v^{i_{b}}(t_{x})-v^{i_{b}}(t_{0})$$

(32) $${\hat{S}}^{i_{b}}(t_{x})-S^{i_{b}}(t_{x})=P^{i_{b}}(t_{x})-P^{i_{b}}(t_{0})-v^{i_{b}}(t_{0})(t_{x}-t_{0})$$

where $P^{i_{b}}(t_{x})-P^{i_{b}}(t_{0})=\int_{t_{0}}^{t_{x}}v^{i_{b}}(t)dt$, and it represents the position variation caused by linear motion $v^{i_{b}}$ during $t_{0}<t<t_{x}$. From Equations (31) and (32), we find that the velocity variation is the influence factor for Algorithm 1, but for Algorithm 2, both the position variation and initial velocity are the influence factors.

Coarse alignment is generally performed under quasi-stationary conditions, and they are characterized as having bounded position and attitude movement such as produced by wind gusts, passengers, and sea waves. The velocity and position variations are bounded, and both of them have same order of magnitude. Unfortunately, for Algorithm 2, the error caused by initial velocity is proportional to time, and this error is bigger than velocity and position variations in general. First, in order to simplify the analysis, the initial velocity is assumed to be compensated for Algorithm 2. After that, the effect of initial velocity is taken into consideration.

According to Equation (21), we have:

(33) $${\left\|\delta C_{i_{b}}^{i}\right\|}_{F}={\left\|G^{-1}\delta F\right\|}_{F}\leq {\left\|G^{-1}\right\|}_{F}\cdot {\left\|\delta F\right\|}_{F}$$

where ${\left\|\cdot \right\|}_{F}$ represents the Frobenious norm,

(34) $${\left\|G^{-1}\right\|}_{F}=\sqrt{\frac{1+{\left\|a^{i}\right\|}_{2}^{2}+{\left\|b^{i}\right\|}_{2}^{2}}{{\left\|c^{i}\right\|}_{2}^{2}}}$$

the detailed derivation of ${\left\|G^{-1}\right\|}_{F}$ can be seen in Appendix A. It is important to notice that the norm of $G^{-1}$ determines the influence of linear motion on error matrix $\delta C_{i_{b}}^{i}$. Evidently, the large norm of $G^{-1}$ leads to amplification of the linear motion influence; in contrast, the small norm of $G^{-1}$ leads to reducing the effect of linear motion on error matrix $\delta C_{i_{b}}^{i}$. Now we consider the two alignment algorithms provided in Section 4, and show how to get the specific expression for matrix norm ${\left\|G^{-1}\right\|}_{F}$ in the following.

#### 5.3.1. Algorithm 1

Let us begin by substituting $V^{i}(t_{k1})$ and $V^{i}(t_{k2})$ into Equation (34), then the norm of matrix $G^{-1}$ can be rewritten as:

(35) $${\left\|G_{V}^{-1}\right\|}_{F}=\sqrt{\frac{1+{\left\|V^{i}(t_{k1})\right\|}_{2}^{2}+{\left\|V^{i}(t_{k2})\right\|}_{2}^{2}}{{\left\|V^{i}(t_{k1})\times V^{i}(t_{k2})\right\|}_{2}^{2}}}$$

In Equation (35), ${\left\|V^{i}(t_{k1})\right\|}_{2}^{2}$ and ${\left\|V^{i}(t_{k2})\right\|}_{2}^{2}$ can be derived from Equation (B4) in Appendix B, and expressed as:

(36) $${\left\|V^{i}(t_{k1})\right\|}_{2}^{2}={\left(\Delta t_{k1}g\right)}^{2}$$

(37) $${\left\|V^{i}(t_{k2})\right\|}_{2}^{2}={\left(\Delta t_{k2}g\right)}^{2}$$

But the norm of $V^{i}(t_{k1})\times V^{i}(t_{k2})$ is difficult to obtain directly. This problem is resolved in a practical manner by using the definition of the cross-product. According to its definition, we have:

(38) $${\left\|V^{i}(t_{k1})\times V^{i}(t_{k2})\right\|}_{2}={\left\|V^{i}(t_{k1})\right\|}_{2}{\left\|V^{i}(t_{k2})\right\|}_{2}\cdot \left|\sin\Theta \right|$$

where Θ is the angle between $V^{i}(t_{k1})$ and $V^{i}(t_{k2})$, and the angular direction is defined by the right hand rule that curls the fingers of the right hand from $V^{i}(t_{k1})$ into $V^{i}(t_{k2})$. Since the coarse alignment is generally accomplished within a short time (only few minutes), we assume that the vectors $-g^{i}(t_{0})$, $V^{i}(t_{k1})$, and $V^{i}(t_{k2})$ lie in the same plane. Then Θ can be obtained by:

(39) $$\Theta =\theta_{V}(t_{k2})-\theta_{V}(t_{k1})$$

where $\theta_{V}(t_{k1})$ is the angle between $-g^{i}(t_{0})$ and $V^{i}(t_{k2})$, $\theta_{V}(t_{k2})$ is the angle between $-g^{i}(t_{0})$ and $V^{i}(t_{k1})$. $\theta_{V}(t_{k1})$ and $\theta_{V}(t_{k2})$ can be drawn from Equation (C4) in Appendix C, and expressed as:

(40) $$\theta_{V}(t_{k1})=\Delta t_{k1}\omega_{ie}\cos L/2$$

(41) $$\theta_{V}(t_{k2})=\Delta t_{k2}\omega_{ie}\cos L/2$$

Thus, Equation (35) transforms into:

(42) $${\left\|G_{V}^{-1}\right\|}_{F}=\sqrt{\frac{1+{\left(\Delta t_{k1}g\right)}^{2}+{\left(\Delta t_{k2}g\right)}^{2}}{{\left(\Delta t_{k1}g\right)}^{2}{\left(\Delta t_{k2}g\right)}^{2}{\left(\Delta t_{k2}-\Delta t_{k1}\right)}^{2}{\left(\omega_{ie}\cos L/2\right)}^{2}}}$$

It is obvious from Equation (42) that the norm of $G^{-1}$ for Algorithm 1 is obtained and determined by parameters $\Delta t_{k1}$, $\Delta t_{k2}$, and L. In other words, the influence of linear motion on error matrix $\delta C_{i_{b}}^{i}$ for Algorithm 1 is determined by parameters $\Delta t_{k1}$, $\Delta t_{k2}$, and L. During the previous derivation, the assumption that the three vectors $-g^{i}(t_{0})$, $V^{i}(t_{k1})$, and $V^{i}(t_{k2})$ lie in the same plane was made in order to simplify the derivation of Θ. Now, for the purpose of validating this assumption, simulations are conducted, and the results are shown in Table 4. The simulation conditions are set as: *t*_0_ = 0 s, *t_k_*_1_ = 50 s, *t_k_*_2_ = 120 s, and the linear velocity caused by the base motion is equal to zero.

**Table 4:** The difference between calculated Θ and actual Θ.

Latitude L	Angle Θ		Error	
	Actual Value	Calculated Value	Absolute Error	Relative Error
0°	0.1462°	0.1462°	0.0000°	0.00%
30°	0.1266°	0.1266°	0.0000°	0.00%
45°	0.1034°	0.1034°	0.0000°	0.00%

Simulation results show that for a short period of time, the difference between calculated Θ and actual Θ is negligible. Therefore, the previous assumption is correct.

#### 5.3.2. Algorithm 2

Similar derivations to those of the last section lead us to get the norm of $G^{-1}$ for Algorithm 2. In this section, ${\left\|S^{i}(t_{k1})\right\|}_{2}^{2}$ and ${\left\|S^{i}(t_{k2})\right\|}_{2}^{2}$ need to be provided, and the assumption that the vectors $-g^{i}(t_{0})$, $S^{i}(t_{k1})$, and $S^{i}(t_{k2})$ lie in the same plane also should be made. Firstly, ${\left\|S^{i}(t_{k1})\right\|}_{2}^{2}$ and ${\left\|S^{i}(t_{k2})\right\|}_{2}^{2}$ are derived from Equation (D3) in Appendix D, and expressed as:

(43) $${\left\|S^{i}(t_{k1})\right\|}_{2}^{2}\;={\left(\Delta t_{k1}^{2}g/2\right)}^{2}$$

(44) $${\left\|S^{i}(t_{k2})\right\|}_{2}^{2}\;={\left(\Delta t_{k2}^{2}g/2\right)}^{2}$$

Secondly, based on the previous assumption, the angle ϒ between $S^{i}(t_{k1})$ and $S^{i}(t_{k2})$ is obtained as:

(45) $$ϒ=\theta_{s}(t_{k2})-\theta_{s}(t_{k1})$$

where $\theta_{S}(t_{k1})$ is the angle between $-g^{i}(t_{0})$ and $S^{i}(t_{k2})$, $\theta_{S}(t_{k2})$ is the angle between $-g^{i}(t_{0})$ and $S^{i}(t_{k1})$. $\theta_{S}(t_{k1})$ and $\theta_{S}(t_{k2})$ can be deduced from Equation (E4) in Appendix E, and described as:

(46) $$\theta_{S}(t_{k1})=\Delta t_{k1}\omega_{ie}\cos L/2\sqrt{2}L/2$$

(47) $$\theta_{S}(t_{k2})=\Delta t_{k2}\omega_{ie}\cos L/2\sqrt{2}L/2$$

Finally, we have:

(48) $${\left\|G_{S}^{-1}\right\|}_{F}=\sqrt{\frac{1+{\left(\Delta t_{k1}^{2}g/2\right)}^{2}+{\left(\Delta t_{k2}^{2}g/2\right)}^{2}}{{\left(\Delta t_{k1}^{2}g/2\right)}^{2}{\left(\Delta t_{k2}^{2}g/2\right)}^{2}{\left(\Delta t_{k2}-\Delta t_{k1}\right)}^{2}{\left(\omega_{ie}\cos L/2\sqrt{2}\right)}^{2}}}$$

Obviously, the specific expression for the norm of $G^{-1}$ can be acquired from Equation (48), and it is also determined by parameters $\Delta t_{k1}$, $\Delta t_{k2}$, and L. Therefore, the influence of linear motion on error matrix $\delta C_{i_{b}}^{i}$ for Algorithm 2 is determined by parameters $\Delta t_{k1}$, $\Delta t_{k2}$, and L, too. In order to validate the assumption made in this section, simulations are performed, and the results are shown in Table 5. The simulation conditions are set as: *t*_0_ = 0 s, *t_k_*_1_ = 50 s, *t_k_*_2_ = 120 s, the linear velocity caused by the base motion is equal to zero.

**Table 5:** The difference between calculated ϒ and actual ϒ.

Latitude L	Angle ϒ		Error	
	Actual Value	Calculated Value	Absolute Error	Relative Error
0°	0.0975°	0.1034°	0.0059°	6.05%
30°	0.0844°	0.0895°	0.0051°	6.04%
45°	0.0689°	0.0731°	0.0042°	6.10%

Obviously, although the calculated value is not equal to the actual value, the difference between them is small. This means that the assumption made in this section is correct as well.

#### 5.3.3. Optimal Parameter Design

From Equations (42) and (48), we find that both of the matrix norms for the two algorithms are determined by parameters $\Delta t_{k1}$, $\Delta t_{k2}$, and L. In operational situations, total integration time $\Delta t_{k2}$ is generally fixed, and the local latitude L is related to the position of the vehicle on which the FOG INS is mounted. Then, however, only parameter $\Delta t_{k1}$ is adjustable. Considering ${\left(\Delta t_{k1}g\right)}^{2}+{\left(\Delta t_{k2}g\right)}^{2}>>1$, ${\left(\Delta t_{k1}^{2}g/2\right)}^{2}+{\left(\Delta t_{k2}^{2}g/2\right)}^{2}>>1$, and by definition,

(49) $$\Delta t_{k2}=\lambda \Delta t_{k1}=T$$

where λ is the ratio between $\Delta t_{k1}$ and $\Delta t_{k2}$, λ>1, and T represents the duration time of coarse alignment. Equations (42) and (48) can be rewritten as:

(50) $${\left\|G_{V}^{-1}\right\|}_{F}=\frac{2}{gT^{2}\omega_{ie}\cos L}\cdot \sqrt{\frac{\lambda^{2}(1+\lambda^{2})}{{\left(\lambda -1\right)}^{2}}}$$

(51) $${\left\|G_{S}^{-1}\right\|}_{F}=\frac{4\sqrt{2}}{gT^{3}\omega_{ie}\cos L}\cdot \sqrt{\frac{\lambda^{2}(1+\lambda^{4})}{{\left(\lambda -1\right)}^{2}}}$$

Evidently, the matrix norms for the two algorithms are determined by parameter λ, and the optimal parameters for the two algorithms are achieved by minimizing the following functions, respectively:

(52) $$f(\lambda )=\sqrt{\frac{\lambda^{2}(1+\lambda^{2})}{{\left(\lambda -1\right)}^{2}}}$$

(53) $$g(\lambda )=\sqrt{\frac{\lambda^{2}(1+\lambda^{4})}{{\left(\lambda -1\right)}^{2}}}$$

Then the determination of optimal parameters is transformed into the solution of the minimum problem. The differential equations for f(λ) and g(λ) are provided as follows:

(54) $$\dot{f}(\lambda )=\sqrt{\frac{{\left(\lambda -1\right)}^{2}}{\lambda^{2}(1+\lambda^{2})}}\cdot \frac{\lambda (\lambda^{3}-2\lambda^{2}-1)}{{\left(\lambda -1\right)}^{3}}$$

(55) $$\dot{g}(\lambda )=\sqrt{\frac{{\left(\lambda -1\right)}^{2}}{\lambda^{2}(1+\lambda^{4})}}\cdot \frac{\lambda (2\lambda^{5}-3\lambda^{4}-1)}{{\left(\lambda -1\right)}^{3}}$$

**Figure 2:**
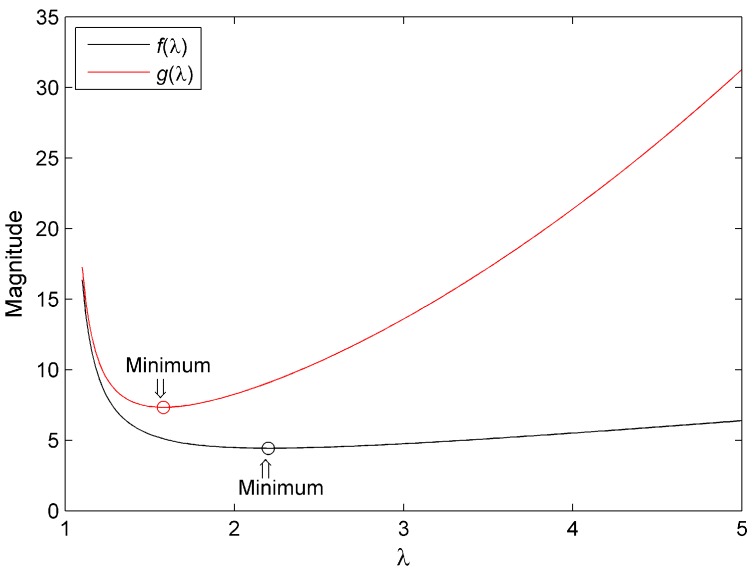
The graphs of functions f(λ) and g(λ).

It is obvious from Equations (54) and (55) that $\dot{f}(\lambda )>0$ and $\dot{g}(\lambda )>0$ in the condition λ>5. Therefore, the minimum will appear on the interval (1, 5). The graphs of functions f(λ) and g(λ) are drawn by Matlab, and shown in Figure 2.

It can be seen from Figure 2 that each of the functions has only one extreme point, and the extreme point is the minimum. Then by solving equations $\dot{f}(\lambda )=0$ and $\dot{g}(\lambda )=0$, the optimal parameters for the two algorithms are obtained as:

(56) $$\lambda_{Vo}=2.20$$

(57) $$\lambda_{So}=1.58$$

The substitution of Equations (56) and (57) into Equations (50) and (51), the optimal norms of $G^{-1}$, can be acquired as:

(58) $${\left\|G_{Vo}^{-1}\right\|}_{F}\approx \frac{8.86}{gT^{2}\omega_{ie}\cos L}$$

(59) $${\left\|G_{So}^{-1}\right\|}_{F}\approx \frac{4.68}{T}\cdot \frac{8.86}{gT^{2}\omega_{ie}\cos L}$$

It is quite obvious that ${\left\|G_{So}^{-1}\right\|}_{F}$ is smaller than ${\left\|G_{Vo}^{-1}\right\|}_{F}$ due to T>>4.68, and this means that the performance of Algorithm 2 is better than that of Algorithm 1 under the disturbance of bounded errors. In order to validate the two optimal parameters provided by Equations (56) and (57), simulations are performed in Simulation C.

Alternately, if the error caused by initial velocity is uncompensated, the performance of Algorithm 2 will be worse than that of Algorithm 1, and a rough and direct explanation for this result is given in the following.

Considering the departure of ${\hat{b}}^{i_{b}}$ from $b^{i_{b}}$ for Algorithm 2, the error caused by initial velocity can be expressed as:

(60) $$v^{i_{b}}(t_{0})(t_{k2}-t_{0})=v^{i_{b}}(t_{0})T$$

Ignoring the departure of ${\hat{a}}^{i_{b}}$ from $a^{i_{b}}$, and then drawing *T* from δF, we have:

(61) $${\left\|G_{So}^{-1}\right\|}_{F}\approx \frac{4.68}{T}\cdot \frac{8.86}{gT^{2}\omega_{ie}\cos L}\cdot T=4.68\cdot \frac{8.86}{gT^{2}\omega_{ie}\cos L}$$

Equation (61) reflects the influence of initial velocity on error matrix $\delta C_{i_{b}}^{i}$ for Algorithm 2. Since the magnitude of initial velocity is close to that of velocity variation, and the value of Equation (61) is bigger than that of Equation (56), we can draw the conclusion that the performance of Algorithm 2 is worse than that of Algorithm 1 while the error produced by initial velocity is uncompensated. This conclusion is verified in simulation D.

Summarizing, Equations (58), (59) and (61) allow us to make the adequate selection of the most accurate algorithm for coarse alignment according to the actual operational conditions. Algorithm 1 is suitable for Marine FOG INS, since the FOG INS is usually disturbed by sea waves and the initial velocity caused by waves is unknown. On the other hand, Algorithm 2 is appropriate for Vehicular FOG INS, because the FOG INS can generally keep stationary at the beginning of the coarse alignment in this operational condition, and then the initial velocity is equal to zero.

## 6. Simulation and Results

### 6.1. Simulation A

To test the effect of sensor errors on the two coarse alignment algorithms, simulations in the stationary base were conducted. In the simulations, an IMU with the following specification is used: FOG error: 0.01°/h and accelerometer error: 10−4 g. The heading, pitch, and roll obey the uniform distribution on the intervals [0,2π], [−π/4,π/4] and [−π/4,π/4], respectively. The local latitude L is set as 45.7796°. The two coarse alignment algorithms are performed simultaneously, and last 120 s. The parameter values of $t_{k1}$ and $t_{k2}$ are set to 50 s and 120 s, respectively. The simulation for the coarse alignment runs six times. In order to show the relationships between the sensor errors and misalignments more explicitly, the direction of the body frame is set to be coincident with that of the navigation frame in the sixth time simulation, *i.e.*, heading 0°, pitch 0°, and roll 0°. The heading, pitch, and roll at the end of the coarse alignments are shown in Table 6, Table 7 and Table 8.

**Table 6:** Alignment results of the six simulations (heading).

	1	2	3	4	5	6
Actual	326.0851°	45.7153°	328.8153°	227.6493°	35.1145°	0°
Algorithm 1	326.1650°	45.7289°	328.8670°	227.6254°	35.0806°	0.0488°
Algorithm 2	326.1650°	45.7289°	328.8670°	227.6254°	35.0807°	0.0489°

**Table 7:** Alignment results of the six simulations (pitch).

	1	2	3	4	5	6
Actual	−19.0352°	4.2193°	41.1756°	41.8400°	−30.8148°	0°
Algorithm 1	−19.9291°	4.2250°	41.1832°	41.8477°	−30.8081°	0.0057°
Algorithm 2	−19.9291°	4.2250°	41.1832°	41.8477°	−30.8081°	0.0057°

**Table 8:** Alignment results of the six simulations (roll).

	1	2	3	4	5	6
Actual	42.3534°	41.1450°	−1.3162°	27.0252°	−32.2302°	0°
Algorithm 1	42.3448°	41.1369°	−1.3237°	27.0149°	−32.2323°	−0.0057°
Algorithm 2	42.3448°	41.1369°	−1.3237°	27.0149°	−32.2323°	−0.0057°

From the alignment results shown in Table 6, Table 7 and Table 8, we can find that little difference exists between Algorithms 1 and 2. It is mainly caused by the calculation error, and can be ignored. That means both of these two coarse alignment algorithms have the same accuracy under the condition of existing internal sensor errors. In the sixth time simulation, since the direction of the body frame is set to be coincident with that of the navigation frame, the form of the DCM calculated by the two coarse alignment algorithms can be unified as:

$${\hat{C}}_{b}^{n}=\left[\begin{array}{ccc} 1 & \phi_{U} & -\phi_{N}\\ -\phi_{U} & 1 & \phi_{E}\\ \phi_{N} & -\phi_{E} & 1 \end{array}\right]$$

Then the relationships between attitude and misalignments can be expressed as:

$$Heading=\arctan(-\phi_{U}/1)\approx -\phi_{U}$$

$$Pitch=\arcsin(-\phi_{E})\approx -\phi_{E}$$

$$Roll=\arctan(-\phi_{N}/1)\approx -\phi_{N}$$

Therefore, from Table 6, Table 7 and Table 8, the misalignments of these two alignment algorithms in the sixth time simulation can be obtained as:

$$\phi_{E}\approx -0.0057{}^{\circ};\;\phi_{N}\approx 0.0057{}^{\circ};\;\phi_{U}\approx -0.0489{}^{\circ}$$

**Figure 3:**
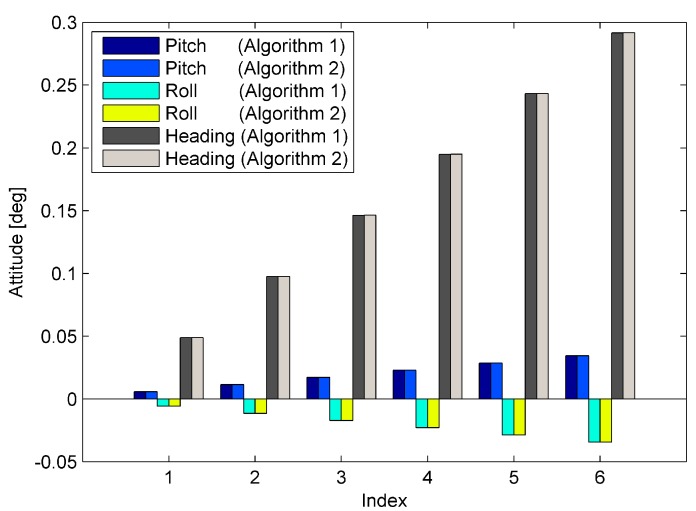
Alignment results of the six simulations.

This result is coincident with Equations (26)–(28), and to some extent, the conclusion that the relationships between alignment errors and sensor errors in the navigation frame can be expressed as Equations (26)–(28) is obtained. In order to validate this conclusion more adequately, six additional simulations were conducted, and the simulation conditions were set as:

Heading 0°, pitch 0°, and roll 0°

$$\text{FOG error: }n\times 0.01{}^{\circ}/h,\text{ accelerometer error: }n\times 10^{-4}g$$

where n∈(1, 2, ⋯, 6) represents the index of the simulations. The results are shown in Figure 3.

It can be seen from Figure 3 that the pitch, roll, and heading are proportional to the magnitudes of the sensor errors. According to the previous analysis, this phenomenon means that the misalignments of these two alignment algorithms are proportional to the magnitudes of the sensor errors. This result corresponds to the relationships represented by Equations (26)–(28), and meanwhile, the results shown in Figure 3 illustrate that both of these two coarse alignment algorithms have the same accuracy under the condition of existing internal sensor errors.

### 6.2. Simulation B

To test the effect of angular motion on the two coarse alignment algorithms, simulations under the condition of angular motion were conducted. In angular movement, the actual heading, pitch, and roll are controlled as:

$$Heading=30{}^{\circ}+5{}^{\circ}\sin(2\pi t/7+\theta_{H})$$

$$Pitch=7{}^{\circ}\sin(2\pi t/5+\theta_{P})$$

$$Roll=10{}^{\circ}\sin(2\pi t/6+\theta_{R})$$

where $\theta_{H}$, $\theta_{P}$, and $\theta_{R}$ obey the uniform distribution on the intervals [0,2π]. The simulation for the coarse alignment runs six times, and their results are shown in Table 9, Table 10 and Table 11.

**Table 9:** Alignment results of the six simulations (heading).

	1	2	3	4	5	6
Actual	25.1507°	33.0046°	32.9933°	25.3219°	3.3252°	28.0762°
Algorithm 1	25.1507°	33.0047°	32.9932°	25.3218°	25.5643°	28.0762°
Algorithm 2	25.1507°	33.0046°	32.9933°	25.3219°	25.5643°	28.0762°

**Table 10:** Alignment results of the six simulations (pitch).

	1	2	3	4	5	6
Actual	0.4499°	4.7596°	−6.6447°	−6.1165°	3.2114°	5.9771°
Algorithm 1	0.4499°	4.7596°	−6.6447°	−6.1165°	3.2114°	5.9771°
Algorithm 2	0.4499°	4.7596°	−6.6447°	−6.1165°	3.2114°	5.9771°

**Table 11:** Alignment results of the six simulations (roll).

	1	2	3	4	5	6
Actual	3.3527°	0.1028°	−6.3304°	−5.1047°	3.3252°	
Algorithm 1	3.3527°	0.1028°	−6.3304°	−5.1047°		8.5160°
Algorithm 2	3.3527°	0.1028°	−6.3304°	−5.1047°	3.3252°	

Obviously, neglecting the little difference between the alignment results and actual values, the attitudes calculated by the two coarse alignment algorithms are the same as the actual one. In other words, these two alignment algorithms are not influenced by angular motion. Thus, the conclusion provided in Section 5.3 is verified.

### 6.3. Simulation C

**Figure 4:**
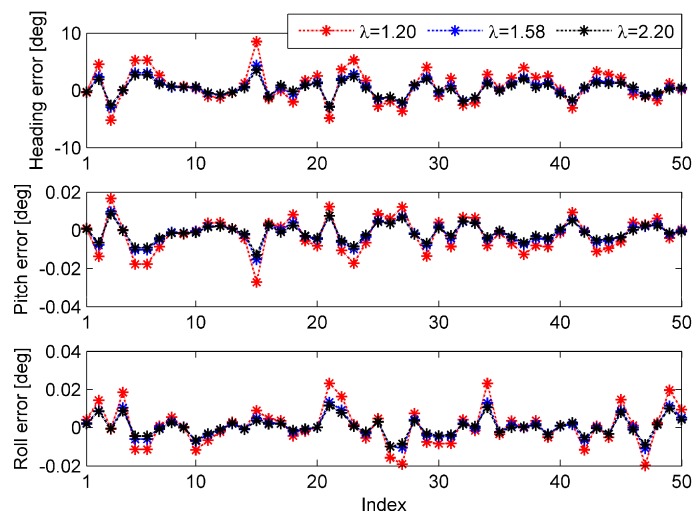
Attitude errors of the 50 simulations for Algorithm 1.

**Figure 5:**
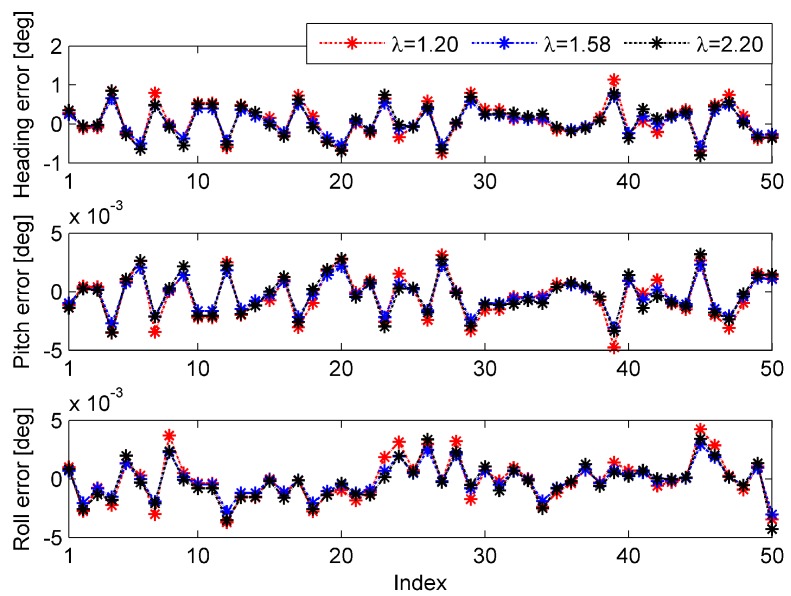
Attitude errors of the 50 simulations for Algorithm 2.

To test the optimal parameters derived in Section 5.3.3, 50 simulations were performed based on the data of the 120 s test for each of the coarse alignment algorithms. Three different parameters were tested in each simulation, and the parameters were set as: λ=1.20,  1.58,  2.20. For Algorithm 1, in order to test the parameters, random velocity variation was introduced into the calculation, and it was modeled as a zero-mean white Gaussian noise of standard deviation 0.02 m/s. Similarly, for Algorithm 2, random position variation was introduced into the calculation, and it was modeled as a zero-mean white Gaussian noise of standard deviation 0.1 m. The simulation results of Algorithm 1 are shown in Figure 4, and their statistics are summarized in Table 12. The results of Algorithm 2 are presented in Figure 5, and their statistics are summarized in Table 13.

**Table 12:** Statistics for Algorithm 1.

Attitude Error [deg]	λ = 1.20		λ = 1.58		λ = 2.20	
	Mean	STD	Mean	STD	Mean	STD
Heading	0.7577	2.7876	0.4008	1.5451	0.3342	1.3773
Pitch	−0.0024	0.0089	−0.0014	0.0051	−0.0012	0.0045
Roll	0.0008	0.0099	0.0004	0.0056	0.0004	0.0049

**Table 13:** Statistics for Algorithm 2.

Attitude Error [deg]	λ = 1.20		λ = 1.58		λ = 2.20	
	Mean	STD	Mean	STD	Mean	STD
Heading	0.0867	0.4489	0.0578	0.3278	0.0666	0.4157
Pitch	−0.0004	0.0019	−0.0002	0.0014	−0.0003	0.0017
Roll	−0.0001	0.0018	−0.0001	0.0013	−0.0002	0.0016

Figure 4 and Table 12 clearly indicate that the performance of Algorithm 1 is determined by the parameter selection. From the simulation results, we can find that λ=2.20 is the adequate selection of the three parameters. Additionally, the tendency of the performance variation to be produced by the parameter variation corresponds to the results of Figure 2. To some extent, the results represented by Figure 4 and Table 12 validate the optimal parameter of Algorithm 1.

Similarly, it can be seen from Figure 5 and Table 13 that the performance of Algorithm 2 is determined by the parameter selection as well. λ=1.58 is the adequate selection of the three parameters, and supports that the trend of the performance variation is produced by the parameter variation,, corresponding to the results of Figure 2. The results represented by Figure 5 and Table 13 partly validate the optimal parameter of Algorithm 2. By comparing the simulation results of Algorithms 1 and 2, we can draw the conclusion that the performance of Algorithm 2 is better than that of Algorithm 1 under the disturbance of bounded errors.

### 6.4. Simulation D

**Figure 6:**
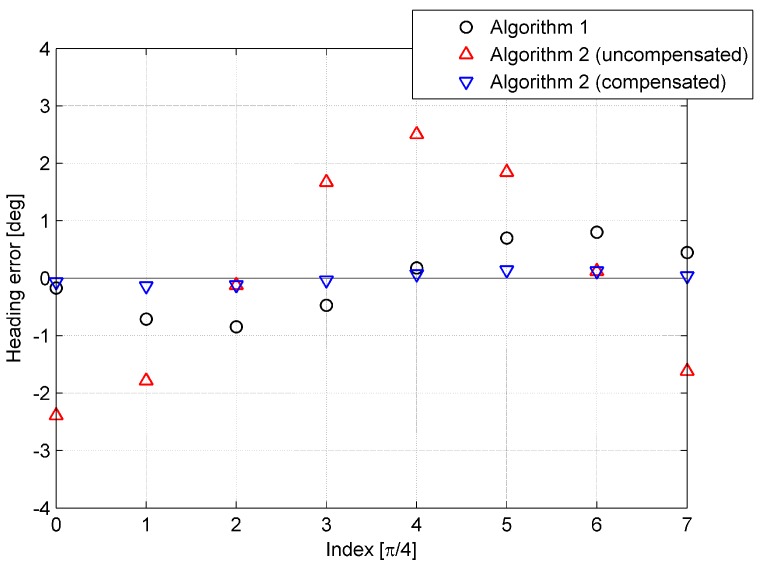
Heading error of the eight simulations.

**Figure 7:**
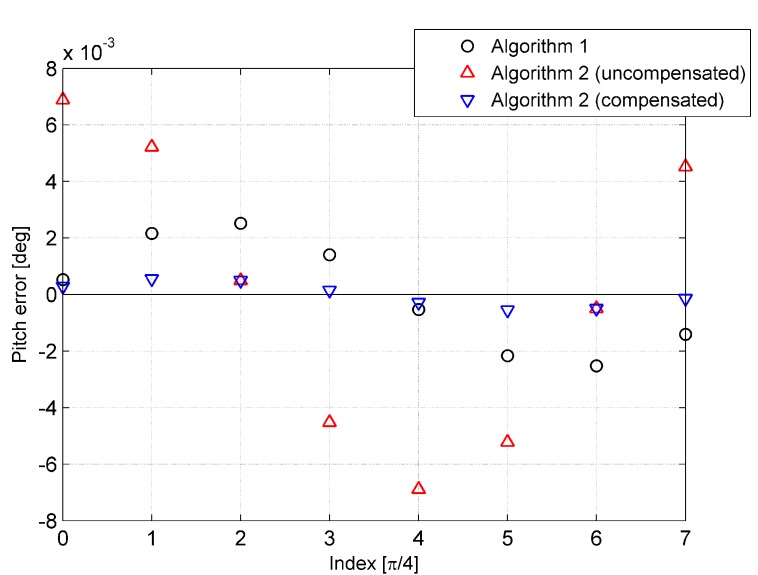
Pitch error of the eight simulations.

**Figure 8:**
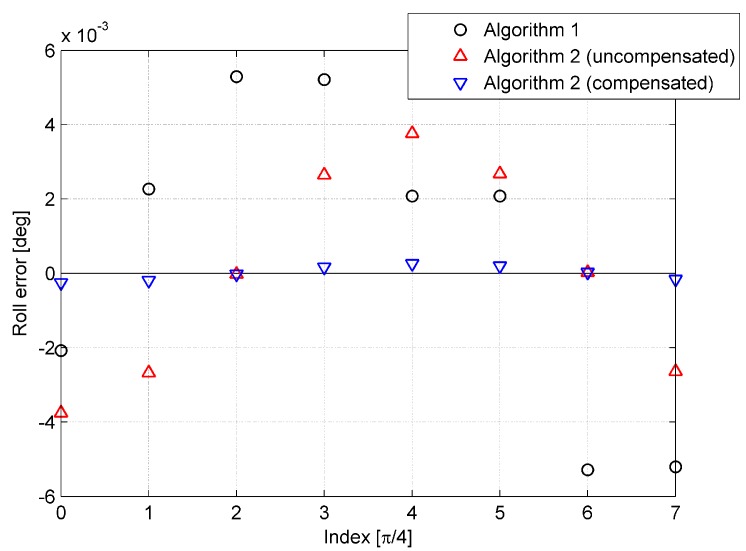
Roll error of the eight simulations.

To test the effect of initial velocity on Algorithm 2, simulations under the condition of linear motion were conducted. The simulation for the coarse alignment ran eight times. In linear movement, the velocities are taken as:

$$v_{x}^{b}=0.02\cos(2\pi t/7+\vartheta )\;\text{m}/\text{s}$$

$$v_{y}^{b}=0.03\cos(2\pi t/6+\vartheta )\;\text{m}/\text{s}$$

$$v_{z}^{b}=0.3\cos(2\pi t/8+\vartheta )\;\text{m}/\text{s}$$

where $v_{x}^{b}$, $v_{y}^{b}$, and $v_{z}^{b}$ represent the components of Earth referenced by velocity V resolved in the body frame b, ϑ is set to nπ/4, and n denotes the index of the simulations, n∈(0, 1, ⋯, 7). The simulation results are provided in Figure 6, Figure 7 and Figure 8.

In each simulation, exclusive of Algorithm 2 where the initial velocity is uncompensated, the simulation in which the initial velocity is compensated was conducted as well. It is obvious from Figure 6, Figure 7 and Figure 8 that the performance of Algorithm 2 is worse than that of Algorithm 1, as the error caused by initial velocity is uncompensated. On the other hand, when the error is compensated, the accuracy of Algorithm 2 is higher than that of Algorithm 1. In Figure 6, Figure 7 and Figure 8, the tendency of the red triangle variation to correspond to the magnitude of initial velocity is shown, and it illustrates that the error caused by initial velocity is the major error of Algorithm 2.

## 7. Experiment and Results

In order to demonstrate the effectiveness of the proposed algorithm, in this section, a lab experiment and a sea experiment were conducted. The lab experiment was implemented in the lab to verify the performance of the proposed algorithm under the stationary base, as the experimental condition was relatively ideal. The data from the sea experiment was used to validate the performance of the proposed algorithm under the base motion.

### 7.1. Lab Experiment

**Figure 9:**
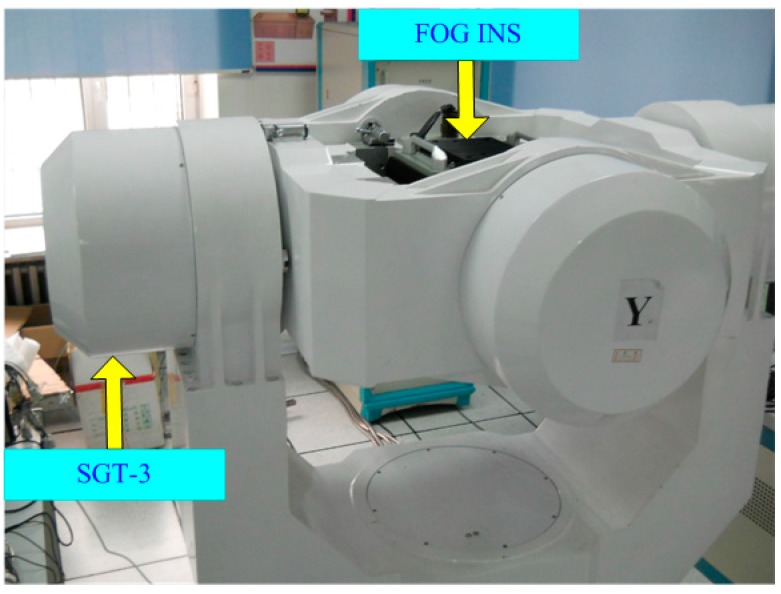
The lab experiment scene.

To test the performance of the proposed algorithm under the stationary base, a lab experiment was conducted. We fixed the FOG INS (FOG bias stability <0.01 °/h, accelerometer bias stability <5 × 10^−5^ g) on the SGT-3 three-axis turntable to implement the alignment experiments. The lab experiment scene can be seen in Figure 9. Eight experiments were implemented. In the experiments, the turntable’s middle and internal gimbal axes lay within the horizontal plane, and the external gimbal axis pointed to upward. The external gimbal angle was set as: $(n\;-\;1)\;\times \;\frac{\pi }{4}$, where n∈(1, 2, ⋯, 8) represents the index of the experiments. Algorithm 1 (λ=2.20, optimal value; λ=2.40, regular value [9]), Algorithm 2 (λ=1.58, optimal value), and fine alignment (gyrocompass alignment method) were performed in each experiment.

In each experiment, the coarse alignment algorithms lasted 120 s, and the fine alignment lasted 20 min, and the results of the fine alignment were used as a reference. The statistics of the heading, pitch, and roll errors are summarized in Table 14. From Table 14, it is clear that the pitch and roll errors of the coarse alignment algorithms are less than 0.0011° (1 σ), and the heading error of the coarse alignment algorithms are less than 0.0038° (1 σ). These errors are basically caused by the sensor noise and turntable control error. Since the heading, pitch, and roll errors are small, the results of the coarse alignment algorithms and fine alignment could be considered equivalent.

**Table 14:** Statistics for the results of the lab experiment.

Attitude Error [deg]	Algorithm 1 (λ=2.20)		Algorithm 1 (λ=2.40)		Algorithm 2 (λ=1.58)	
	Mean	STD	Mean	STD	Mean	STD
Heading	0.0015	0.0017	0.0018	0.0016	0.0017	0.0021
Pitch	−0.0005	0.0005	−0.0005	0.0004	−0.0004	0.0004
Roll	0.0004	0.0003	0.0003	0.0006	0.0004	0.0007

### 7.2. Sea Experiment

**Figure 10:**
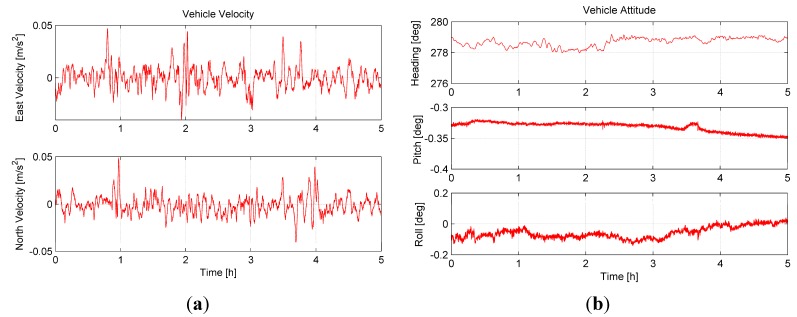
(**a**) The velocity of the vehicle; (**b**) the attitude of the vehicle.

To test the performance of the proposed algorithm under the base motion, a sea experiment was conducted in the South Sea of China. In the experiment, the ship was under the mooring condition. A FOG INS was used for the experiment, the attitude reference was given by the Photonic Inertial Navigation System (PHINS) from the company iXBlue. The velocity and attitude of the ship shown in Figure 10 are provided by the PHINS. Fifty experiments were implemented. In the experiments, the start time of coarse alignment was set as: (n − 1) × 0.1 h, where n∈(1, 2, ⋯, 50) represents the index of the experiments. Algorithm 1 (λ=2.20, optimal value; λ=2.40, regular value [9]), and Algorithm 2 ( λ=1.58, optimal value) were performed in each experiment.

In each experiment, the coarse alignment algorithms lasted 120 s. The statistics of the heading, pitch, and roll errors are summarized in Table 15. Owing to the existence of the installation errors between the FOG INS and PHINS, the mean of the fifty experiments could only be used to estimate the installation errors. The performance of the coarse alignment algorithms is reflected by the STD. From Table 15, we can find that the performance of Algorithm 1 (λ=2.20, optimal value) is the best, and the performance of Algorithm 2 (λ=1.58, optimal value) is the worst. This result demonstrate the analysis made in this paper.

**Table 15:** Statistics for the results of the sea experiment.

Attitude Error [deg]	Algorithm 1 (λ = 2.20)		Algorithm 1 (λ = 2.40)		Algorithm 2 (λ = 1.58)	
	Mean	STD	Mean	STD	Mean	STD
Heading	0.0752	0.1321	0.0729	0.1431	0.0807	0.1699
Pitch	0.3089	0.0013	0.3089	0.0013	0.3086	0.0015
Roll	−0.1162	0.0008	−0.1162	0.0008	−0.1162	0.0009

## 8. Conclusions

In this paper, two coarse alignment algorithms based on the inertial reference frame are introduced and analyzed. Particularly, the analysis of the effect of sensor errors and base motion on coarse alignment is made. Based on the analysis made in this paper, the following meaningful conclusions have been acquired:

- Both of these two coarse alignment algorithms have the same accuracy under the condition of existing internal sensor errors.
- In the stationary base, the misalignments of the algorithms are identical to those obtained with fine alignment methods.
- The optimal ratio between $\Delta t_{k1}$ and $\Delta t_{k2}$ exists. For Algorithm 1, it is equal to 2.20, but for Algorithm 2, it is equivalent to 1.58.
- Under the disturbance of linear motion, the performance of Algorithm 2 will be better than that of Algorithm 1 if the error caused by initial velocity is compensated. Otherwise, the accuracy of Algorithm 2 will be worse than that of Algorithm 1.

Simulation results have validated the above conclusions. Based on the above conclusions, we also conclude that:

- Algorithm 1 is suitable for Marine FOG INS;
- Algorithm 2 is appropriate to be used for Vehicular FOG INS.

Moreover, the results of the lab and sea experiments demonstrate the effectiveness of the proposed algorithms.

## Appendix

### A. The Derivation of ${\left\|G^{-1}\right\|}_{F}$

It is well known that the inverse matrix $G^{-1}$ can be expressed by the following equation:

(A1) $$G^{-1}=\frac{G^{*}}{\left|G\right|}$$

where $G^{*}$ is the adjoint matrix of matrix G, |G| represents the determinant of matrix G.

From Equations (3), (4) and (7), the elements of matrix $G^{*}$ can be obtained as:

$$g_{11}^{*}=b_{y}(a_{x}b_{y}-a_{y}b_{x})-b_{z}(a_{z}b_{x}-a_{x}b_{z})$$

$$g_{12}^{*}=a_{z}(a_{z}b_{x}-a_{x}b_{z})-a_{y}(a_{x}b_{y}-a_{y}b_{x})$$

$$g_{13}^{*}=a_{y}b_{z}-a_{z}b_{y}$$

$$g_{21}^{*}=b_{z}(a_{y}b_{z}-a_{z}b_{y})-b_{x}(a_{x}b_{y}-a_{y}b_{x})$$

$$g_{22}^{*}=a_{x}(a_{x}b_{y}-a_{y}b_{x})-a_{z}(a_{y}b_{z}-a_{z}b_{y})$$

$$g_{23}^{*}=a_{z}b_{x}-a_{x}b_{z}$$

$$g_{31}^{*}=b_{x}(a_{z}b_{x}-a_{x}b_{z})-b_{y}(a_{y}b_{z}-a_{z}b_{y})$$

$$g_{32}^{*}=a_{y}(a_{y}b_{z}-a_{z}b_{y})-a_{x}(a_{z}b_{x}-a_{x}b_{z})$$

$$g_{33}^{*}=a_{x}b_{y}-a_{y}b_{x}$$

Hence,

(A2) $$\begin{array}{l} {\left\|G^{*}\right\|}_{F}^{2}=\sum_{i=1}^{3}\sum_{j=1}^{3}(g_{ij}^{*}{)}^{2}\\ \;=(1+\sum_{i=x,y,z}a_{i}^{2}+\sum_{i=x,y,z}b_{i}^{2})\cdot [{\left(a_{x}b_{y}-a_{y}b_{x}\right)}^{2}+{\left(a_{y}b_{z}-a_{z}b_{y}\right)}^{2}+{\left(a_{z}b_{x}-a_{x}b_{z}\right)}^{2}]\\ \;=(1+{\left\|a^{i}\right\|}_{2}^{2}+{\left\|b^{i}\right\|}_{2}^{2})\cdot {\left\|c^{i}\right\|}_{2}^{2} \end{array}$$

where ${\left\|\cdot \right\|}_{2}$ denotes the vector norm. On the other hand, ${\left|G\right|}^{2}$ can be expressed as:

(A3) $$\begin{array}{l} {\left|G\right|}^{2}={\left[{\left(a_{x}b_{y}-a_{y}b_{x}\right)}^{2}+{\left(a_{y}b_{z}-a_{z}b_{y}\right)}^{2}+{\left(a_{z}b_{x}-a_{x}b_{z}\right)}^{2}\right]}^{2}\\ \;={\left\|c^{i}\right\|}_{2}^{4} \end{array}$$

Consequently,

(A4) $${\left\|G^{-1}\right\|}_{F}=\sqrt{\frac{{\left\|G^{*}\right\|}_{F}^{2}}{{\left|G\right|}^{2}}}=\sqrt{\frac{1+{\left\|a^{i}\right\|}_{2}^{2}+{\left\|b^{i}\right\|}_{2}^{2}}{{\left\|c^{i}\right\|}_{2}^{2}}}$$

### B. The Derivation of ${\left\|V^{i}(t)\right\|}_{2}^{2}$

After the alignment process gets start, the gravity vector in the inertial frame can be expressed as follows:

(B1) $$g^{i}(t)=\left[\begin{array}{c} -g\cos L\cos\omega_{ie}(t-t_{0})\\ -g\cos L\sin\omega_{ie}(t-t_{0})\\ -g\sin L \end{array}\right]$$

The velocity of strapdown INS in the inertial frame is defined as:

(B2) $$V^{i}(t)=-\int_{t_{0}}^{t}g^{i}(t)dt=\left[\begin{array}{c} g\cos L\sin\omega_{ie}\Delta t/\omega_{ie}\\ g\cos L(1-\cos\omega_{ie}\Delta t)/\omega_{ie}\\ \Delta tg\sin L \end{array}\right]$$

where $\Delta t=t-t_{0}$. Then,

(B3) $$\begin{array}{l} {\left\|V^{i}(t)\right\|}_{2}^{2}={\left(g\cos L\sin\omega_{ie}\Delta t/\omega_{ie}\right)}^{2}+{\left(g\cos L(1-\cos\omega_{ie}\Delta t)/\omega_{ie}\right)}^{2}+{\left(\Delta tg\sin L\right)}^{2}\\ \;={\left(g\cos L/\omega_{ie}\right)}^{2}\cdot 2(1-\cos\omega_{ie}\Delta t)+{\left(\Delta tg\sin L\right)}^{2}\\ \;={\left(g\cos L/\omega_{ie}\right)}^{2}\cdot 4\sin^{2}(\omega_{ie}\Delta t/2)+{\left(\Delta tg\sin L\right)}^{2} \end{array}$$

Let us simplify at this point by using the first-order approximation for the sine function in Equation (B3), namely

$$\sin(\omega_{ie}\Delta t/2)\approx \omega_{ie}\Delta t/2$$

Therefore, we have:

(B4) $${\left\|V^{i}(t)\right\|}_{2}^{2}\approx {\left(\Delta tg\cos L\right)}^{2}+{\left(\Delta tg\sin L\right)}^{2}={\left(\Delta tg\right)}^{2}$$

### C. The Angle $\theta_{v}(t)$ between Vectors $-g^{i}(t_{0})$ and $V^{i}(t)$

The connection between cosine function of $\theta_{V}(t)$ and vectors $-g^{i}(t_{0})$, $V^{i}(t)$ is described as:

(C1) $$\cos\theta_{V}(t)=\frac{-g^{i}(t_{0})\cdot V^{i}(t)}{\left\|-g^{i}(t_{0})\right\|\left\|V^{i}(t)\right\|}$$

From Equations (B1) and (B2), we have:

(C2) $$\begin{array}{l} -g^{i}(t_{0})\cdot V^{i}(t)={\left(g\cos L\right)}^{2}\sin\omega_{ie}\Delta t/\omega_{ie}+{\left(g\sin L\right)}^{2}\Delta t\\ \;=2{\left(g\cos L\right)}^{2}\sin(\omega_{ie}\Delta t/2)(1-2\sin^{2}(\omega_{ie}\Delta t/4))/\omega_{ie}+{\left(g\sin L\right)}^{2}\Delta t \end{array}$$

Substituting Equations (B1), (B4) and (C2) into Equation (C1), and by considering the approximation $\sin(\omega_{ie}\Delta t/2)\approx \omega_{ie}\Delta t/2$, $\cos\theta_{V}(t)$ becomes:

(C3) $$\begin{array}{l} \cos\theta_{V}(t)\approx 1-2\cos^{2}L\sin^{2}(\omega_{ie}\Delta t/4)\approx 1-2\sin^{2}(\Delta t\omega_{ie}\cos L/4)\\ \;=\cos(\Delta t\omega_{ie}\cos L/2) \end{array}$$

Therefore, $\theta_{V}(t)$ can be obtained as:

(C4) $$\theta_{V}(t)=\Delta t\omega_{ie}\cos L/2$$

### D. The Derivation of ${\left\|S^{i}(t)\right\|}_{2}^{2}$

The position of strapdown INS in the inertial frame is defined as follows:

(D1) $$S^{i}(t)=\int_{t_{0}}^{t}V^{i}(t)dt=\left[\begin{array}{c} g\cos L(1-\cos\omega_{ie}\Delta t)/\omega_{ie}^{2}\\ g\cos L(\omega_{ie}\Delta t-\sin\omega_{ie}\Delta t)/\omega_{ie}^{2}\\ \Delta t^{2}g\sin L/2 \end{array}\right]$$

Hence, we would have:

(D2) $$\begin{array}{l} {\left\|S^{i}(t)\right\|}_{2}^{2}={\left(g\cos L(1-\cos\omega_{ie}\Delta t)/\omega_{ie}^{2}\right)}^{2}+\\ \;{\left(g\cos L(\omega_{ie}\Delta t-\sin\omega_{ie}\Delta t)/\omega_{ie}^{2}\right)}^{2}+{\left(\Delta t^{2}g\sin L/2\right)}^{2}\\ \;={\left(g\cos L/\omega_{ie}^{2}\right)}^{2}\cdot \;[4\sin^{2}(\omega_{ie}\Delta t/2)+{\left(\omega_{ie}\Delta t\right)}^{2}-\\ \;4\omega_{ie}\Delta t\sin(\omega_{ie}\Delta t/2)(1-2\sin^{2}(\omega_{ie}\Delta t/4))]+{\left(\Delta t^{2}g\sin L/2\right)}^{2}\; \end{array}$$

Take the following approximation into consideration:

$$\sin(\omega_{ie}\Delta t/2)\approx \omega_{ie}\Delta t/2$$

$$\sin(\omega_{ie}\Delta t/4)\approx \omega_{ie}\Delta t/4$$

Finally, we can obtain:

(D3) $$\begin{array}{l} {\left\|S^{i}(t)\right\|}_{2}^{2}\;\approx \;{\left(g\cos L/\omega_{ie}^{2}\right)}^{2}\cdot [4{\left(\omega_{ie}\Delta t/2\right)}^{2}+{\left(\omega_{ie}\Delta t\right)}^{2}-\\ \;2{\left(\omega_{ie}\Delta t\right)}^{2}(1-2{\left(\omega_{ie}\Delta t/4\right)}^{2})]+{\left(\Delta t^{2}g\sin L/2\right)}^{2}\\ \;={\left(\Delta t^{2}g\cos L/2\right)}^{2}+{\left(\Delta t^{2}g\sin L/2\right)}^{2}={\left(\Delta t^{2}g/2\right)}^{2} \end{array}$$

### E. The Angle $\theta_{S}(t)$ between Vectors $-g^{i}(t_{0})$ and $S^{i}(t)$

The cosine function of $\theta_{S}(t)$ can be represented as:

(E1) $$\cos\theta_{S}(t)=\frac{-g^{i}(t_{0})\cdot S^{i}(t)}{\left\|-g^{i}(t_{0})\right\|\left\|S^{i}(t)\right\|}$$

From Equations (B1) and (D1), we can obtain:

(E2) $$\begin{array}{l} -g^{i}(t_{0})\cdot S^{i}(t)={\left(g\cos L/\omega_{ie}\right)}^{2}(1-\cos\omega_{ie}\Delta t)+{\left(\Delta tg\sin L\right)}^{2}/2\\ \;=8{\left(g\cos L/\omega_{ie}\right)}^{2}\sin^{2}(\omega_{ie}\Delta t/4)(1-\sin^{2}(\omega_{ie}\Delta t/4))+\;{\left(\Delta tg\sin L\right)}^{2}/2 \end{array}$$

Hence, by taking the approximation $\sin(\omega_{ie}\Delta t/4)\approx \omega_{ie}\Delta t/4$ into consideration, $\cos\theta_{S}(t)$ is given by:

(E3) $$\begin{array}{l} \cos\theta_{S}(t)\approx 1-{\left(\cos L\right)}^{2}\sin^{2}(\omega_{ie}\Delta t/4)\approx 1-2\sin^{2}(\Delta t\omega_{ie}\cos L/4\sqrt{2})\\ \;=\cos(\Delta t\omega_{ie}\cos L/2\sqrt{2}) \end{array}$$

Finally, we could have:

(E4) $$\theta_{S}(t)=\Delta t\omega_{ie}\cos L/2\sqrt{2}$$
